# Variable sea‐ice conditions influence trophic dynamics in an Arctic community of marine top predators

**DOI:** 10.1002/ece3.5313

**Published:** 2019-05-30

**Authors:** Isabeau Pratte, Birgit M. Braune, Keith A. Hobson, Mark L. Mallory

**Affiliations:** ^1^ Biology Acadia University Wolfville Nova Scotia Canada; ^2^ Environment and Climate Change Canada, National Wildlife Research Centre Carleton University Ottawa Ontario Canada; ^3^ Environment and Climate Change Canada, Department of Biology University of Western Ontario London UK

**Keywords:** high Arctic, prebreeding, seabirds, stable isotopes

## Abstract

Sea‐ice coverage is a key abiotic driver of annual environmental conditions in Arctic marine ecosystems and could be a major factor affecting seabird trophic dynamics. Using stable isotope ratios of carbon (δ^13^C) and nitrogen (δ^15^N) in eggs of thick‐billed murres (*Uria lomvia*), northern fulmars (*Fulmarus glacialis*), glaucous gulls (*Larus hyperboreus*), and black‐legged kittiwakes (*Rissa tridactyla*), we investigated the trophic ecology of prebreeding seabirds nesting at Prince Leopold Island, Nunavut, and its relationship with sea‐ice conditions. The seabird community of Prince Leopold Island had a broader isotopic niche during lower sea‐ice conditions, thus having a more divergent diet, while the opposite was observed during years with more extensive sea‐ice conditions. Species' trophic position was influenced by sea ice; in years of lower sea‐ice concentration, gulls and kittiwakes foraged at higher trophic levels while the opposite was observed for murres and fulmars. For murres and fulmars over a longer time series, there was no evidence of the effect of sea‐ice concentration on species' isotopic niche. Results suggest a high degree of adaptation in populations of high Arctic species that cope with harsh and unpredictable conditions. Such different responses of the community isotopic niche also show that the effect of variable sea‐ice conditions, despite being subtle at the species level, might have larger implications when considering the trophic ecology of the larger seabird community. Species‐specific responses in foraging patterns, in particular trophic position in relation to sea ice, are critical to understanding effects of ecosystem change predicted for a changing climate.

## 1 INTRODUCTION

Sea ice and its extensive presence throughout the spring and summer characterize polar seas of both hemispheres. Sea ice is fundamental to the cycle of biomass abundance in Arctic marine ecosystems, and its annual phenology of development and breakup plays a dominant role in ecosystem structure and function (Michel, Ingram, & Harris, 2006; Piepenburg, 2005; Post et al., 2013). Sea ice is critical for ice‐obligated and ice‐associated animals that rely heavily on its surfaces to forage or reproduce (Bradstreet & Cross, 1982; Gilg et al., 2016; Laidre et al., 2015; Moore & Huntington, 2008). However, decades of fluctuation in melt and freeze‐up dates, pack extent, and less predictable floe dynamics (as a result of climate change) threaten the stability of ice‐based communities (Markus et al., 2009; Post et al., 2013). Loss of sea ice and its reduced predictability will lead to colonization by species from southern regions and range contractions for ice‐dependent species (Forcada & Trathan, 2009; Post et al., 2013; Vihtakari et al., 2018).

Sea‐ice condition around colonies of Arctic‐breeding seabirds plays a major role in determining annual breeding phenology and success (Emmerson & Southwell, 2008; Gaston Gilchrist & Hipfner, 2005; Gaston Gilchrist & Mallory, 2005; Love, Gilchrist, Descamps, Semeniuk, & Bêty, 2010; Mallory & Forbes, 2007; Prop et al., 2015). In addition, sea ice could be a major factor affecting seabird trophic dynamics within a community. For Arctic predators like seabirds, foraging on zooplankton (e.g., *Calanus* spp., *Parathemisto* spp.), and fish (e.g., Arctic cod *Boreogadus saida*), sea ice is an essential factor contributing to the highly productive food web on which they rely. The reductions in or absence of sea ice induced by earlier breakup of the pack could decouple trophic linkages between primary and secondary producers in the food web (Michel et al., 2006; Piepenburg, 2005; Post et al., 2013) and change the structure of the planktonic community in the Arctic (Eisner, Napp, Mier, Pinchuk, & Andrews, 2014; Fujiwara, Hirawake, Suzuki, Imai, & Saitoh, 2014). Such changes potentially alter the abundance and access to preferred prey for seabirds (Divoky, Lukacs, & Drucknmiller, 2015; Gaston, Gilchrist, Mallory, & Smith, 2009; Provencher, Gaston, O'Hara, & Gilchrist, 2012). Loss of sea ice could thus induce seabirds to forage on suboptimal prey of lower energy content, which could have adverse effects on breeding success and fitness (Divoky et al., 2015; Gaston, Gilchrist, & Hipfner, 2005).

Arctic seabirds require open water to forage and also associate with sea‐ice margins and edges (Bradstreet & Cross, 1982; Mehlum & Gabrielsen, 1993), and thus, they are good models to study the effect different sea‐ice conditions can have on the trophic dynamics of species within a community. Increased intraspecific competitive interaction in response to limited access to open water, long flying distances to the floe edge, changes in food web structure, or mismatches with timing of peak availability of preferred prey, could favor the expansion of the species' dietary niche.

We investigated the trophic and isotopic ecology of prebreeding female northern fulmar (*Fulmarus glacialis*), thick‐billed murre (*Uria lomvia*), black‐legged kittiwake (*Rissa tridactyla*), and glaucous gull (*Larus hyperboreus*) at Prince Leopold Island (PLI), Nunavut, a colony of major importance for both diversity and abundance of seabirds in the Canadian high Arctic. The prelaying period at the breeding site is a critical time for Arctic‐breeding migratory seabirds, as the females are principally income breeders, acquiring energy and nutrients required for egg production from local prey resources (e.g., Mallory, Forbes, Ankney, & Alisauskas, 2008, Jacobs, Elliott, Gaston, & Weber, 2009). During that period, the pack ice that covers the Arctic Ocean in the long winter months has started its annual breakup, leading to the formation of channels, leads, and areas of open water that vary in size and dynamics between years. The PLI colony is located near a recurring ice edge that varies in position from year to year (Gaston, Gilchrist, & Hipfner, 2005). In some years, sea‐ice breaks up early, and no pack ice remains around the colony by the end of June. In other years, ice cover in adjacent waters breaks up later than usual, so that during the prelaying period open water is far from the colony causing seabirds to invest more time and energy to access suitable foraging areas (Bradstreet, 1980; Gaston, Gilchrist, & Hipfner, 2005; Gaston, Gilchrist, & Mallory, 2005).

Sea‐ice conditions near PLI have been associated with changes in trophic position of the mentioned species, each one responding differently to high or low sea‐ice years, presumably reflecting their specific foraging behavior (Moody, Hobson, & Gaston, 2012). Seabirds in this community belong to the same functional group of marine top predators as indicated previously by stable isotope analyses (Hobson & Welch, 1992a). However, each species selects a range of different prey items (Byers, Smith, & Mallory, 2010; Hobson, 1993; Lønne & Gabrielsen, 1992; Mallory et al., 2012) and thus tends to occupy different trophic positions and has a specific trophic ecology that varies even throughout the year (Hobson & Bond, 2012). For example, murres are surface divers and prey notably on small fish (Arctic cod, capelin *Mallotus villosus*) and invertebrates such as euphausiids (Gaston & Nettleship, 1981). Kittiwakes and gulls are opportunistic and can only access the surface layer of the water column for prey such as euphausiids, amphipods, and schooling fish (capelin, Arctic cod), but glaucous gulls also prey upon other seabird eggs and chicks (Hatch, Robertson, & Herron Baird, 2009; Weiser & Gilchrist, 2012). Northern fulmars are known to capture small squid, crustaceans (mostly copepods), and polychaete worms (Byers et al., 2010). Our overall objective was to examine the trophic ecology of the seabird community of PLI during the prebreeding season under different sea‐ice conditions using stable isotope measurements in egg tissues.

Starting in the late 1970s, northern fulmar and thick‐billed murre eggs were collected at PLI as part of a contaminants monitoring program (Mallory & Braune, 2012). The isotopic values for both nitrogen (δ^15^N) and carbon (δ^13^C) in those eggs were measured since the late 1990s. These metrics are suitable proxies of trophic position and some aspects of foraging location (e.g., pelagic vs. inshore) for birds during egg formation (Hobson & Welch, 1992a, Hobson, 1993, see also Hupp, Ward, Soto, & Hobson, 2018). For 3 years during the monitoring period, black‐legged kittiwake and glaucous gull isotopic values in eggs were also obtained. We used these data to address whether sea‐ice conditions influenced the isotopic niche area and trophic position of female seabirds during prebreeding, a key period to acquire energy for principal income breeders (Hobson, Sirois, & Gloutney, 2000; Mallory et al., 2008; Sénéchal, Bêty, Gilchrist, Hobson, & Jamieson, 2011). Given the importance of sea ice in Arctic marine food web composition and structure, and for high Arctic seabirds (Divoky et al., 2015; Moody et al., 2012), we predicted (a) low‐ice years would be associated with broader species isotopic niches as a response to limited access to preferred prey types that associate with ice and (b) this response would vary among species because of their specific trophic ecology. For example, we expected northern fulmar would show little variation in both trophic position (Moody et al., 2012) and niche size, mainly because of its limited access to deeper layers of the water column (but see Hobson & Welch, 1992b), and its ability to fly to distant and predictably open water (Mallory et al., 2008). Thus, we predicted that, at the community level and during years of lower ice conditions, species' isotopic niches would be more divergent and community niche space would widen, particularly in response to reduced availability of ice‐associated prey, such as Arctic cod, a preferred prey item (e.g., Gaston & Hipfner, 2000). To test those predictions, we examined isotopic data from four species obtained during 3 years of markedly different sea‐ice conditions and also assessed the relationship between sea‐ice concentration and isotopic niche over a decade of data collected for northern fulmars and thick‐billed murres. We aimed to provide insight into the direct influence sea‐ice conditions had on the trophic dynamics of a community of Arctic marine top predators and so provide new insight into the importance of seabirds as indicators of ocean forage conditions in the high Arctic.

## 2 METHODS

### 2.1 Data collection

We collected eggs during the last week of June or first week of July at Prince Leopold Island (PLI; 74.01°N, 90.02°W), Nunavut. Eggs were collected from active nests in 1998, 2003, and 2005–2015 for northern fulmars, and 2003 and 2005–2014 for thick‐billed murres. Black‐legged kittiwake and glaucous gull eggs were also collected in 2003, 2008, and 2013. We did not include black guillemot (*Cepphus grylle*) in this study because eggs of that species were not collected during the same 3 years as for black‐legged kittiwake and glaucous gull. Fifteen eggs were collected each year with the following exceptions: 2003—12 black‐legged kittiwake eggs; 2008 and 2013—nine and 12 glaucous gull eggs, respectively; 2015—six northern fulmar eggs.

Eggs were kept cool in the field and shipped to the National Wildlife Research Centre (NWRC), Ottawa, Ontario, for processing and chemical analyses (detailed in Braune, Gaston, & Mallory, 2019). Egg contents were homogenized and stored frozen (−40°C) in acid‐rinsed polyethylene vials.

### 2.2 Isotopic analyses

Stable isotope analyses for the samples collected in 1998–2011 were conducted at the Department of Soil Science, University of Saskatchewan, Saskatoon, Saskatchewan, whereas samples collected in 2012–2015 were analyzed at the University of Ottawa G.G. Hatch Stable Isotope Laboratory, Ottawa, Ontario. We prepared egg homogenates by freeze‐drying them, grinding them to powder, and then removing lipids using a 2:1 chloroform:methanol soak and rinse. We performed isotope assays on 1‐mg subsamples of homogenized material loaded into tin cups.

The 1998–2011 samples were analyzed on a Europa 20:20 continuous‐flow isotope ratio mass spectrometer (CFIRMS) interfaced with a Robo‐Prep elemental analyzer. Within each analytical run, five unknowns were separated by two albumen laboratory standards. The 2012–2015 samples were analyzed using an Isotope Cube Elemental Analyser (Elementar) interfaced with a Delta Advantage continuous‐flow isotope ratio mass spectrometer (Thermo) using a ConFlo III (Thermo). A glutamic acid laboratory standard was included for every 10 unknown samples. Quality control was maintained by running sample duplicates. All measurements are reported in standard δ‐notation in parts per thousand (‰) relative to the AIR international standard. Replicate measurements of internal laboratory standards [1998–2011 samples: albumen, 2012–2014 samples: C‐55 (glutamic acid)] indicated measurement errors of ±0.3‰ and ±0.2‰, respectively. Interlaboratory comparisons of duplicate samples (*n* = 45) were consistent within measurement error; that is, mean values for δ^15^N between the two laboratories differed by <0.2‰ and a *t* test comparison of results for the duplicate samples indicated no significant difference (*p > *0.05).

Strong linear negative relationships between δ^13^C values and C:N ratios for each species indicated high interyear variability in the efficiency of lipid extraction from the samples. We, therefore, normalized the δ^13^C values relative to samples that had complete lipid removal based on the lowest C:N ratio measured for each species and which was within the theoretical range for completely lipid‐free samples (see Post et al., 2007 for rationale). We assumed that the lowest egg homogenate C:N ratio for each species was representative of lipid‐free samples and used the derived regression equations for ∆^13^C (i.e., with lipid–no lipid content) versus C:N to normalize the measured δ^13^C values:

$$\begin{array}{cc} \text{Murre}:\;\Delta^{13}C=1.656-0.430*\text{C:N} & r^{2}=0.71 \end{array}$$

$$\begin{array}{cc} \text{Fulmar}:\Delta^{13}C=2.202-0.624*\text{C:N} & r^{2}=0.93 \end{array}$$

$$\begin{array}{cc} \text{Kittiwake:}\;\Delta^{13}C=2.415-0.622*\text{C:N} & r^{2}=0.79 \end{array}$$

$$\begin{array}{cc} \text{Gull}:\Delta^{13}C=2.345-0.686*\text{C:N} & r^{2}=0.88 \end{array}$$

As lipids contain negligible nitrogen, as expected, there was no relationship between egg δ^15^N and C:N for any of the species, so the δ^15^N values did not require correction.

### 2.3 Data analysis

**Figure 1:**
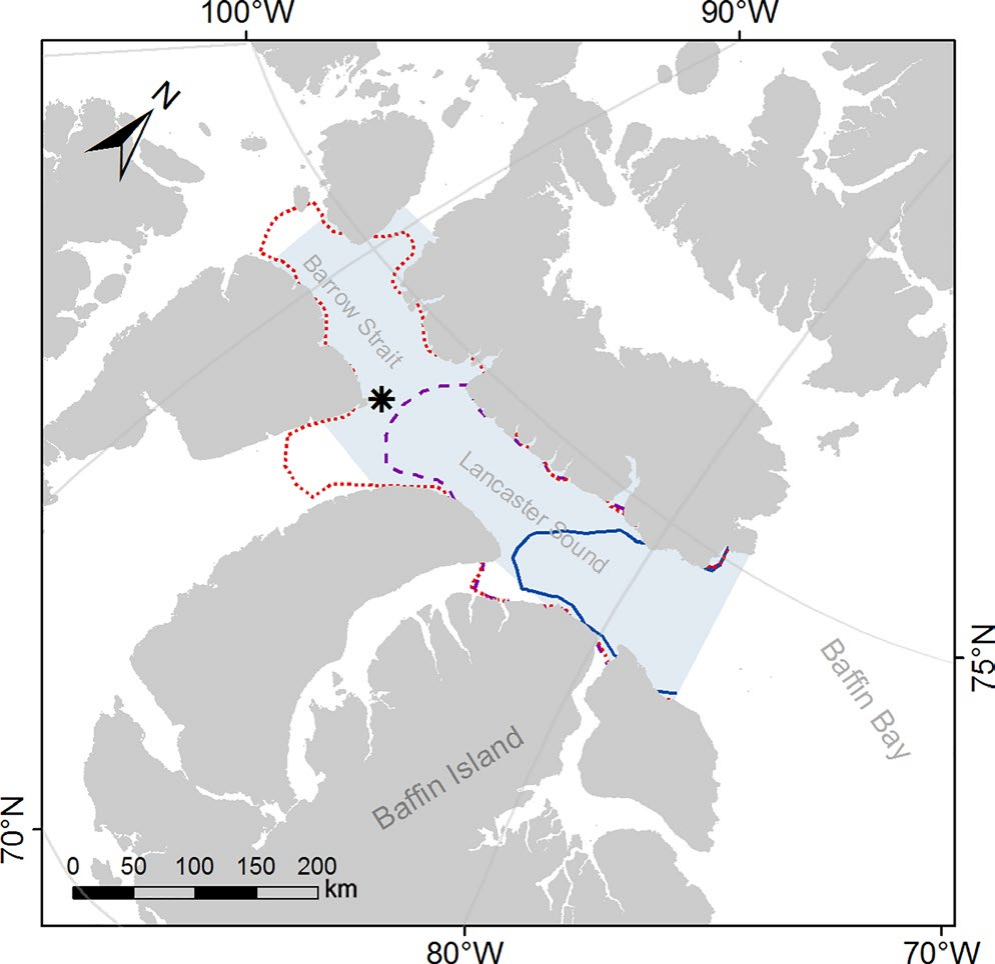
Lancaster Sound and part of Barrow Strait area (shaded) in Nunavut, from which the sea‐ice concentration data were extracted. The black star locates the seabird colony of Prince Leopold Island; the sea‐ice extent (floe edge) in June at 70% concentration is presented for 2008—blue solid line, 2013—purple dashed line, and 2003—red dotted line. The ice concentrations in the shaded area were, respectively for those 3 years, 67%, 45%, and 21%

We downloaded monthly average sea‐ice concentration data derived from satellite Nimbus‐7 SMMR and DMSP SSM/I‐SSMIS Passive Microwave at a grid cell size of 25 × 25 km (Cavalieri, Parkinson, Gloersen, & Zwally, 1996). Those datasets can be found on the National Snow and Ice Data Center (NSIDC) website https://nsidc.org/data/NSIDC-0051/versions/1. The data were imported in ArcGIS (ESRI, 2012), and for each year, we extracted the average sea‐ice concentration (%) in May and June for an area including Lancaster Sound and a section of Barrow Strait (Figure 1). This area represents the main marine habitat used by seabirds nesting at PLI (Nettleship & Gaston, 1978). The sea ice in May represented the conditions encountered during the prelaying period for the northern fulmar, whereas June represented the prelaying period for thick‐billed murre and black‐legged kittiwake, with glaucous gulls falling intermediate to these groups (Gaston & Hipfner, 2000; Mallory et al., 2012).

The species studied are mostly income breeders (Jacobs et al., 2009; Mallory et al., 2008; Moe et al., 2009), in that most nutrients transferred to the egg by the female would have been acquired locally in their Arctic‐breeding grounds. To calculate the trophic position (TP) of each species studied, we used the following equation:

$${\text{TP}}_{\text{consumer}}=\lambda +\frac{\left(\delta^{15}N_{\text{consumer}}-\delta^{15}N_{\text{base}}\right)}{\Delta \delta_{\text{dt}}}$$

where TP_consumer_ is the trophic position of the consumer, ∆δ_dt_ is the average diet—tissue discrimination factor, δ^15^N_conusmer_ is that of the consumer's egg (‰), and δ^15^N_base _is that of the primary consumer or primary herbivore (TP = 2) at the base of the food chain. Here, we used a mean δ^15^N of 7.8‰ for *Calanus hyperboreus* sampled in Lancaster Sound (Pomerleau et al., 2011) for δ^15^N_base_. λ is the trophic position of the base: assumed to be 2.0 for *C. hyperboreus* (primary consumer, Hobson & Welch, 1992a). For ∆δ_dt_, we used a value based on average discrimination factors of 3.5‰ for yolk and 3.1‰ for albumen (i.e., peregrine falcon *Falco peregrinus* mean discrimination factor between diet‐albumen and diet‐lipid‐free yolk of captive birds fed ad libidum and thus assumed income breeder [Hobson, 1995]). We then corrected these discrimination factors according to the yolk and albumen mass for each species based on egg content values obtained in the literature. The details of the egg content percentages and final discrimination factor used for each species are presented in Table 1.

**Table 1:** Egg content of the four species based on literature

	Mass_whole egg_	Mass_yolk_	Mass_albumen_	Species source	References	% Lipids_whole egg_	Mass_lipid‐free yolk_	% Yolk_lipid free_	% Albumen	Discrimination factor_whole egg_ (‰)
Black‐legged kittiwake^a^	108.44	28.63	71.90	Glaucous gull	Verboven, Verreault, Letcher, Gabrielsen, & Evans, 2009	8.5	18.98	21	79	3.19
Glaucous gull^a^	108.44	28.63	71.90	Glaucous gull	Verboven et al., 2009	8.9	18.98	21	79	3.19
Northern fulmar^b^	103.4	30.50	61.6	Northern fulmar	Warham, 1983	10.5	19.64	24	76	3.20
Thick‐billed murre^b^	111.85	36.40	61.20	Thick‐billed murre	Hipfner, Gaston, Herzberg, Brosnan, & Storey, 2003	12.1	22.80	27	73	3.21

Notes

Values of glaucous gull were used as proxy for black‐legged kittiwake since no egg content data were found for that species in the literature. Following this equation ${\text{Mass}}_{\text{lipid - free yolk}}={\text{Mass}}_{\text{yolk}}-\left({\text{Mass}}_{\text{yolk}}\times \frac{\%{\text{lipids}}_{\text{whole egg}}}{\%{\text{yolk}}_{\text{whole egg}}}\right),$ we then calculated the percentage of yolk_lipid free_ and adjusted the average discrimination factor of 3.5‰ for yolk and 3.1‰ for albumen to a discrimination factor for the whole egg (yolk and albumen).

a The yolk mass was lipid‐adjusted based on whole egg lipid percentages reported in Braune, Donaldson, and Hobson (2002).

b The yolk mass was lipid‐adjusted based on whole egg lipid percentages of pooled eggs collected at our site between 2003 and 2015.

We determined the isotopic niche of northern fulmar and thick‐billed murre using Stable Isotope Bayesian Ellipses in R—the “SIBER” package (Jackson, Inger, Parnell, & Bearhop, 2011; R Core Team, 2017). We used a probabilistic method (Jackson et al., 2011) to calculate the mode and credible interval (Cr.I.) of Bayesian‐simulated Standard Ellipse Areas (SEA*_b_*) for each species each year, based on a posteriori distribution of standard ellipses obtained following 10,000 iterations. In addition to the northern fulmar and thick‐billed murre, we estimated using the same probabilistic method the isotopic niche of the black‐legged kittiwake and glaucous gull in 3 years during which eggs of the four species were collected: 2003, 2008, and 2013. To better infer the effect of various sea‐ice conditions on isotopic niche at the community level, we also used a Bayesian probabilistic method (Jackson et al., 2011) to compare two relevant community metrics proposed by Layman, Arrington, Montaña, and Post (2007) across the 3 years of different sea‐ice conditions. We used nearest neighbor distance (NND; low NND indicates clustering of the species and trophic redundancy) and the Euclidian distance to centroid (CD; indicating the average trophic diversity of the community). We also calculated the community niche space using convex hull fitted over the species means (total area [TA]); a measure less sensitive to outliers than a regular convex hull fitted over all samples (Layman et al., 2007; Jackson et al., 2011). We tested the difference in the community metrics (NND, CD, TA) across years of different ice conditions using the Bayesian probabilistic method described above (a posteriori distribution of the metrics following 10,000 iterations). Based on this Bayesian approach, the probability *p_b_* corresponds to the number of iterations for a group (paired species and year) SEA*_b_* that are smaller (or greater) than the number of iterations for the compared group SEA*_b_*:

$$p_{b}=\frac{\sum ({\text{SEA}}_{b1}<{\text{SEA}}_{b2})}{\text{total number iterations}}$$

We calculated the average distance to the floe edge, which we defined as the 70% sea‐ice concentration in June and used that parameter along with the overall sea‐ice concentration in the Barrow Strait—Lancaster Sound area to determine the ice condition in 2003, 2008, and 2013. Based on those parameters, we considered 2003 as a “Low” ice year, 2008 as an “Extensive” ice year, and 2013 as a “Moderate” ice year (21%, 67% and 45% ice concentration, respectively; Figure 1). We tested the significance of sea ice condition, species, and their interaction on the trophic position using generalized linear model (GLM) followed with likelihood ratio test (*F*‐statistic) to look at the significance of the fixed terms.

**Figure 2:**
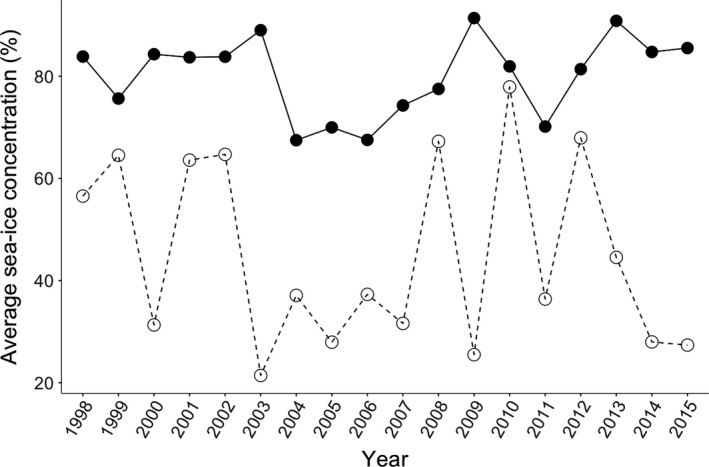
Average sea ice concentration (%) in May (black circles) and June (open circles) for the Barrow Strait—Lancaster Sound area, NU, from 1998 to 2015

Given the availability of multiple years of egg isotopic data for the northern fulmar and thick‐billed murre, we tested for the fixed effect of average sea‐ice concentration (%) in Lancaster Sound in interaction with species and species alone on the annual mode of isotopic niche (SEA*_b_*) and on the annual mean trophic position of both species using again GLM followed with likelihood ratio test (*F*‐statistic) to look at the significance of the fixed terms. We chose to add the interaction between average sea‐ice concentration and species in our models since the period of the prelaying season differs between the species leading to a biological interaction a priori between species and sea‐ice concentration since sea ice is more extensive in May (northern fulmar prelaying period) than in June (thick‐billed murre prelaying period; Figure 2).

## 3 RESULTS

**Figure 3:**
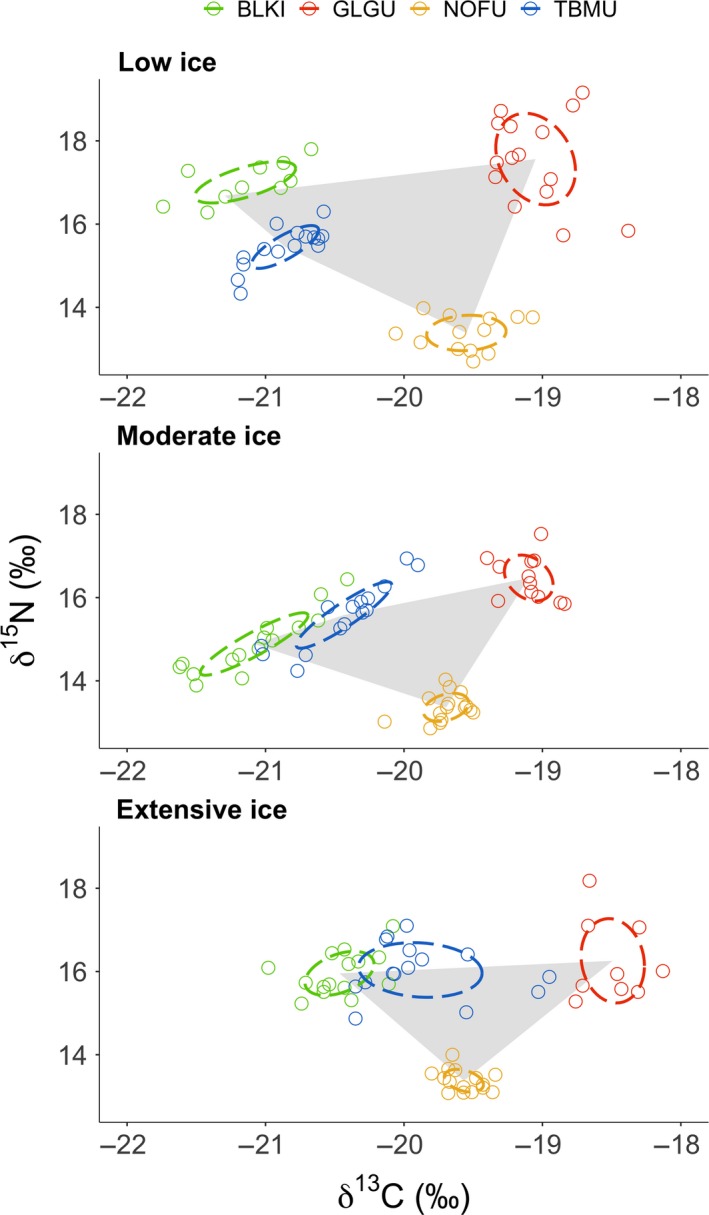
Standard ellipses corrected for small sample size (dashed line; 40% credible interval; Jackson & Parnell, 2011) determined from stable isotope values in eggs of black‐legged kittiwakes (BLKI), glaucous gulls (GLGU), Northern fulmars (NOFU), and thick‐billed murres (TBMU). Also presented are the community niche space as convex hull area based on each species means of δ^13^C and δ^15^N values for 3 years representing low (2003), moderate (2013), and extensive (2008) sea‐ice conditions in Barrow Strait—Lancaster Sound area, NU

**Figure 4:**
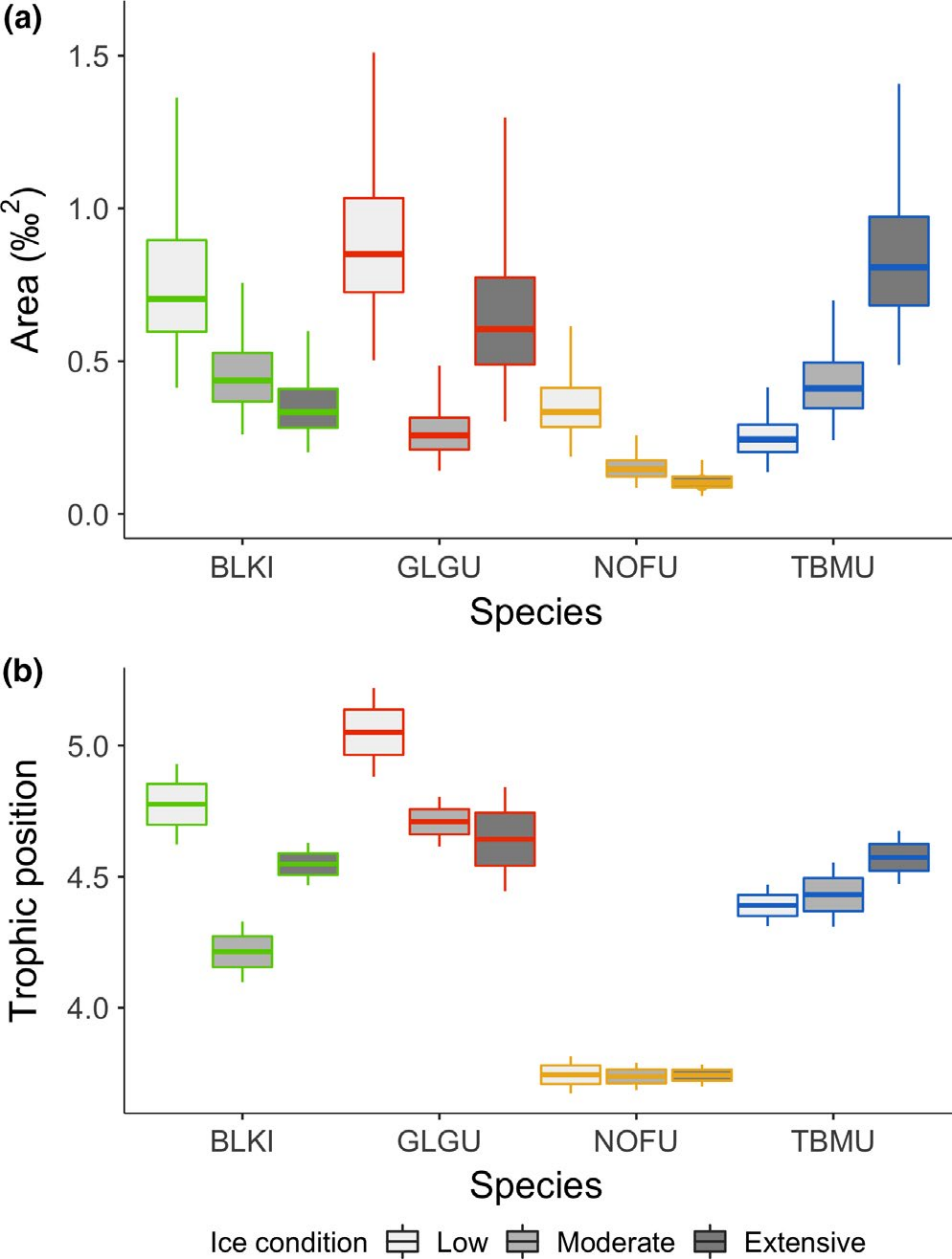
(a) Mode of isotopic niche width (‰^2^) with 50% and 95% credible intervals obtained following posterior estimates of Bayesian Standard Ellipse Areas (SEAb; Jackson & Parnell, 2011), and (b) mean (± SE and 95% CI) trophic position based on δ^15^N values from eggs of black‐legged kittiwake (BLKI), glaucous gull (GLGU), Northern fulmar (NOFU), and thick‐billed murre (TBMU) collected at Prince Leopold Island, NU, in 3 years of different sea‐ice condition: 2003—low, 2013—moderate, and 2008—extensive sea ice

**Figure 5:**
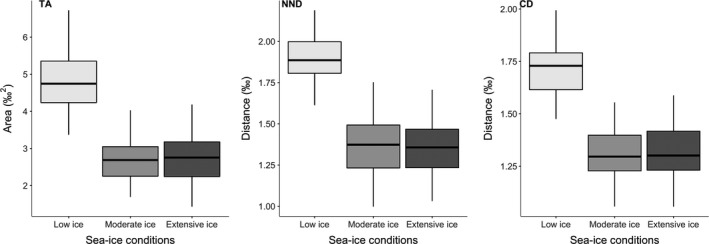
Boxplots depicting the Bayesian posterior estimates of the community isotopic niche (TA), the distance between species (NND), and the distance to centroid of species (CD). Mode, 50%, and 95% credible intervals are presented; overlap indicates the degree of similarity for the different estimates of community niche area between years of various sea‐ice conditions

Isotopic niches of the four species did not overlap in low and moderate ice years, but thick‐billed murre and black‐legged kittiwake clusters overlapped during the extensive ice year 2008 (8%; Figure 3). Following the Bayesian posterior distribution of estimated SEA*_b_*, both black‐legged kittiwakes and glaucous gulls had broader isotopic niches than thick‐billed murres during the low‐ice year 2003 (both; *p_b_* < 0.01; Figure 4a). Black‐legged kittiwakes showed broader isotopic niches in that year compared with 2008, the year of extensive ice conditions (*p_b_* = 0.03). In contrast, thick‐billed murre isotopic niche was broader during the year of extensive sea ice than in the two other years (both; *p_b_* < 0.03). The difference between low and extensive ice years was not as marked for glaucous gulls for which the isotopic niche was significantly smaller in the year of moderate ice conditions compared with the two other years (both; *p_b_* < 0.02; Figure 4a). Fulmars had a small SEA*_b_* with the smallest variation (Cr.I.) of the four species (Table 2). The seabird community isotopic niche, assessed following a posteriori estimates of TA (convex hull), was broader in 2003 compared with the two other years (both; *p_b_* < 0.03), while 2008 and 2013 had similar community isotopic niche areas (Figure 5). This meant that the overall isotopic niche occupied by the seabird community during prebreeding at PLI broadened under lower sea‐ice conditions. Accordingly, distance between the species' niches (NND) was greater during low‐ice conditions compared with moderate and heavier ice years (both; *p_b_* < 0.02; Figure 5) indicating lower trophic redundancy among species. The average degree of trophic diversity (CD) was also larger in 2003 compared with the two other years of heavier sea‐ice conditions (both; *p_b_* < 0.02, Figure 5), likely influenced by the larger niche width of black‐legged kittiwake, glaucous gull and northern fulmar that year (above; Figure 3). Thus, all community metrics indicated that the species trophic ecology was more divergent that year (Figures 3 and 5) compared with the more clustered species niches under heavier ice conditions.

**Table 2:** Mean ± *SD* δ^13^C and δ^15^N values (in ‰), C:N ratio, trophic position (TP), and mode of isotopic niche area (95% credible interval—Cr.I.) following Bayesian posterior estimates of Standard Ellipse Area (SEA_b_) for black‐legged kittiwake, glaucous gull, northern fulmar, and thick‐billed murre eggs collected at Prince Leopold Island, NU in three distinct years of sea‐ice conditions: 2003, 2008, and 2013, respectively, “low,” “extensive,” and “moderate” ice years

Species	Ice condition	δ^13^C	δ^15^N	C:*N*	TP	*n*	SEA_b_ (95% Cr.I.)
Black‐legged kittiwake	Low	−21.29 ± 0.47	16.68 ± 0.87	7.62 ± 0.49	4.77 ± 0.27	12	0.71 (0.39–1.73)
	Moderate	−21.08 ± 0.34	14.89 ± 0.73	3.63 ± 0.02	4.21 ± 0.22	15	0.44 (0.25–0.78)
	Extensive	−20.47 ± 0.24	15.95 ± 0.51	4.26 ± 0.22	4.55 ± 0.16	15	0.33 (0.20–0.60)
Glaucous gull	Low	−19.73 ± 0.28	17.56 ± 1.07	7.27 ± 0.47	5.05 ± 0.33	15	0.84 (0.50–1.53)
	Moderate	−19.10 ± 0.17	16.47 ± 0.53	3.53 ± 0.06	4.71 ± 0.17	12	0.26 (0.14–0.46)
	Extensive	−18.49 ± 0.22	16.26 ± 0.97	4.04 ± 0.12	4.64 ± 0.30	9	0.63 (0.28–1.31)
Northern fulmar	Low	−19.53 ± 0.27	13.48 ± 0.54	7,99 ± 0.50	3.75 ± 0.13	13	0.44 (0.23–0.76)
	Moderate	−19.70 ± 0.16	13.36 ± 0.33	3.51 ± 0.04	3.74 ± 0.10	15	0.17 (0.09–0.25)
	Extensive	−19.57 ± 0.14	13.38 ± 0.26	4.13 ± 0.14	3.74 ± 0.08	15	0.10 (0.06–0.17)
Thick‐billed murre	Low	−20.86 ± 0.23	15.45 ± 0.50	8.72 ± 0.43	4.39 ± 0.16	15	0.24 (0.14–0.42)
	Moderate	−20.43 ± 0.34	15.58 ± 0.78	3.53 ± 0.02	4.43 ± 0.24	15	0.42 (0.24–0.73)
	Extensive	−19.88 ± 0.43	16.03 ± 0.64	4.70 ± 0.32	4.57 ± 0.20	15	0.79 (0.49–1.42)

Trophic position varied across species and was influenced by sea‐ice condition (*F*_6,165_ = 9.27, *p* < 0.01); black‐legged kittiwakes and glaucous gulls occupied a higher trophic position in 2003, the year of low ice, compared with the other 2 years (Figure 4b). Thick‐billed murres displayed an opposite trend; their trophic position was higher in the year of extensive ice cover (Table 2). Fulmars were the most consistent of the species, maintaining the lowest trophic position in the 3 years, with less variation in trophic position annually (mean coefficient of variation 2.8%) compared with kittiwakes (3.7%), gulls (5.0%), or murres (6.1%).

**Figure 6:**
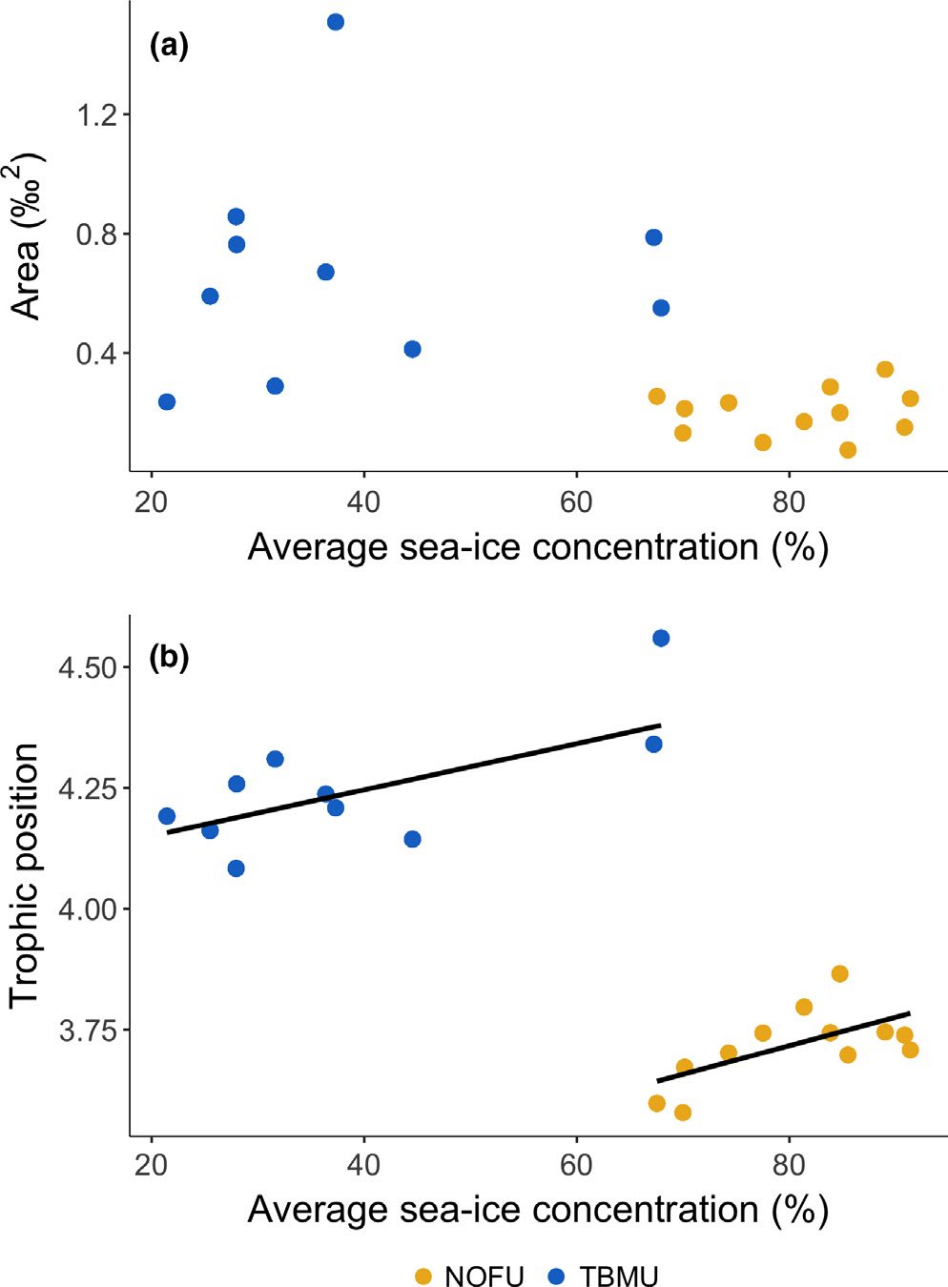
Relationship between average sea‐ice concentrations (%) in Barrow Strait—Lancaster Sound area and (a) the mode of isotopic niche area (‰^2^) obtained following posterior estimates of Bayesian Standard Ellipse Areas (SEA_b_), and (b) the annual mean trophic position each year calculated from northern fulmar (NOFU) and thick‐billed murre (TBMU) eggs collected at Prince Leopold Island, NU, between 1998 and 2015 (2003–2014 for thick‐billed murre). Fifteen eggs were collected each year, except in 2015 during which 6 eggs were collected for northern fulmar only, and in 2003 only 13 eggs were used in the analyses for northern fulmar due to two outliers. Trend line represents significant relationship

**Figure 7:**
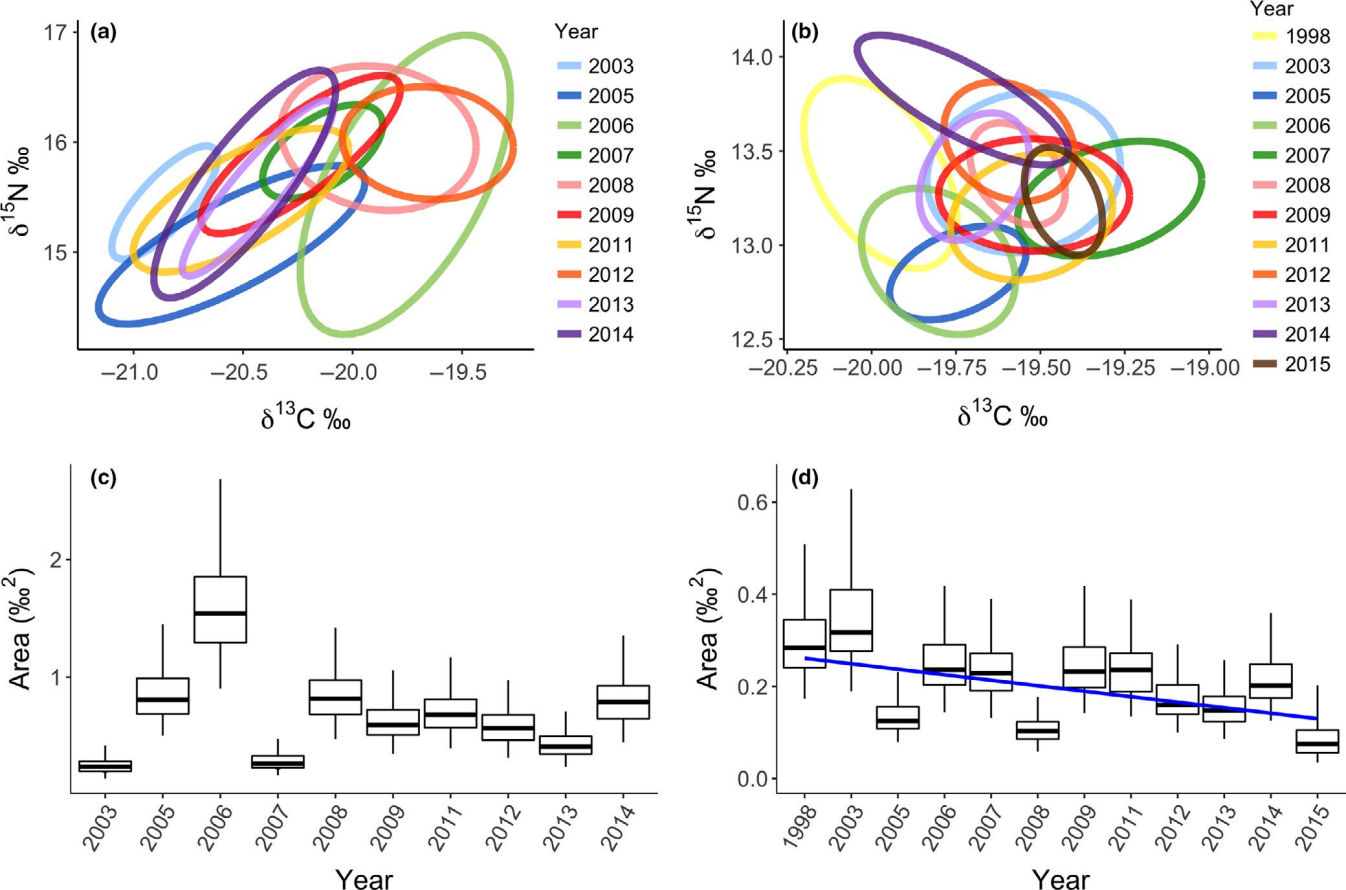
Isotopic niche widths represented by standard ellipses corrected for small sample size (40% credible interval; Jackson & Parnell, 2011) determined from stable isotope values in eggs of thick‐billed murre (TBMU; a) and northern fulmar (NOFU; b) collected at Prince Leopold Island, NU, over 10 and 12 years (respectively). Also presented are boxplots of the Bayesian posterior estimates of Standard Ellipse Area (SEA_b_) in each year for TBMU (c) and NOFU (d). Mode, 50%, and 95% credible intervals are presented; overlap of the boxes indicates the degree of similarity for the different isotopic niche width between years for each species. Trend line represents significant linear relationship between years and the mode of isotopic niche area. Fifteen eggs were collected each year, except in 2015 during which 6 eggs were collected for northern fulmar only, and in 2003 only 13 eggs were used in the analyses for northern fulmar due to two outliers

Between 1998 and 2015, there was no significant temporal trend in sea‐ice concentration in Barrow Strait—Lancaster Sound area for either May (*r*_18_ = 0.14, *p* = 0.57) or June (*r*_18_ = 0.20, *p* = 0.45; Figure 2). During this time, the northern fulmar and the thick‐billed murre did not exhibit any significant relationship between average sea‐ice concentrations and their isotopic niche (*F*_3,18_ = 0.16, *p* = 0.86; Figure 6a). Overall, the thick‐billed murre had a broader isotopic niche than the northern fulmar (*F*_3,18_ = 17.16, *p* < 0.01). The average trophic position of both thick‐billed murres and northern fulmars was positively influenced by average sea‐ice concentration (Figure 6b), that is, trophic position of murres and fulmars increased with higher concentration of ice (*F*_3,18_ = 8.80, *p* = 0.002), as suggested for the thick‐billed murre in the four species model (Figure 3b). Note that neither murre (*F*_1,8_ = 0.99, *p* = 0.35) nor fulmar (*F*_1,10_ = 1.03, *p* = 0.33) trophic position exhibited a significant trend through time. We did not find any significant temporal trend in thick‐billed murre isotopic niche (*F*_1,8_ = 0.05, *p* = 0.83), although fulmar isotopic niche slightly narrowed over time (*F*_1,10_ = 6.36, *p* = 0.03) (Figure 7c,d).

## 4 DISCUSSION

Arctic seabirds are long‐lived and their colonies resilient under a range of annual conditions (e.g., Gaston & Nettleship, 1981, Grémillet et al., 2012, Grémillet et al. 2015), and some of which are driven by long‐term climate cycles (e.g., Irons et al., 2008). Increasingly, research has documented the effects of directional patterns of warmer temperatures and reduced sea‐ice cover on Arctic seabirds. These effects include earlier breeding, increased parasite load, reduced breeding success, and reduced survival (Descamps, Aars, et al., 2017; Gaston, Gilchrist, & Hipfner, 2005; Gaston, Gilchrist, & Mallory, 2005). Evidence is accumulating that changing sea‐ice dynamics and sea‐surface temperatures have altered both the timing of peak abundance and the types of prey near seabird colonies (Doney et al., 2012; Gaston, Woo, & Hipfner, 2003; Grémillet et al., 2012; Renner et al., 2016). In this study, we provide novel indicators of facultative responses in the trophic ecology of a high Arctic seabird community relative to different sea‐ice regimes, which may portend future conditions under a changing climate.

Adaptations for seabirds living sympatrically in an environment constrained by access to food resources, both temporally and spatially, have likely led to divergence in behaviors that limit overlap in species' trophic ecology (Linnebjerg et al., 2013; Navarro et al., 2013; Pratte, Robertson, & Mallory, 2017; Robertson et al., 2014). All four species in our study have varied diets (Gaston & Hipfner, 2000; Hatch et al., 2009; Mallory et al., 2010; Weiser & Gilchrist, 2012) and generally occupied distinct isotopic niches. Under all sea‐ice conditions, species also maintained their relative trophic position; glaucous gulls always fed higher in the trophic web while northern fulmars consistently foraged at the lowest trophic position (see also Hobson & Welch, 1992a, Hobson & Welch, 1992b).

We had predicted that during low‐ice years, limited abundance of preferred prey would be reflected in broader species niches and thus community isotopic niches. This was confirmed; we found that during the low‐ice year, the community isotopic niche was broader, presumably a result of the increased distance among species' niches that were also broader that year except for thick‐billed murre. When preferred forage prey is abundant, most individuals are predicted to use the same resource, leading to low population‐level isotopic variance (i.e., narrow isotopic niche area; Hobson, Piatt, & Pitocchelli, 1994, Yeakel, Bhat, Elliott Smith, & Newsome, 2015). Considering interspecific interaction, increased distance between species isotopic niches has been associated with preferred prey limitation and nutritional stress in seabirds (e.g., thick‐billed and common murre—*Uria aalge*; Barger & Kitaysky, 2012). Although thick‐billed murres had a narrower isotopic niche when sea ice was low, the other three species had broader niches, and overall divergence increased among species leading to broader community isotopic niche.

Changes in the food web following the absence of sea ice have the potential of limiting the abundance and availability of preferred prey like Arctic cod (Divoky et al., 2015; Provencher et al., 2012). Following recent decreases in sea‐ice extent, changes in key forage prey have been observed in Arctic seabirds (e.g., black guillemot, thick‐billed murre), which switched diet from mostly Arctic cod to prey like sculpin or capelin that are not as ice‐associated (Divoky et al., 2015; Provencher et al., 2012). Similar switches in fish species have not yet been clearly distinguished in the diet of thick‐billed murre chicks at the high Arctic PLI colony, likely attributable to the typically extensive presence of sea ice in the region (Provencher et al., 2012). Nonetheless, during the low‐ice year, we observed broader niches at the species level for kittiwake, glaucous gull, and fulmar (i.e., they were using a larger array of prey resources), suggesting a greater influence of individual foraging specializations or increased generalist strategies in response to a diversified isotopic landscape. In contrast, increased clustering between thick‐billed murre and black‐legged kittiwake isotopic niches led to a narrower community isotopic niche under heavier sea‐ice conditions. These two contrasting responses suggest that sea‐ice conditions likely influence the prey isotopic spectrum available to seabirds. Few studies have looked at trophic, community‐wide responses in relation to abiotic factors affecting the trophic ecology of seabirds. Thus, responsive patterns like the increased community isotopic niche we observed under low‐ice conditions could reflect the limited availability and access to key forage prey, underlining the importance of having a community‐scale approach to confirm and better understand how changes in sea‐ice conditions are affecting organisms.

In contrast to Moody et al. (2012) who sampled incubating birds at the same colony, we found that the trophic positions of prebreeding gulls and kittiwakes were higher in years of low‐ice cover compared with heavier ice years. These annual differences in trophic positions suggest that either the individuals foraged on organisms occupying higher trophic levels in the food web that year (i.e., different species or age classes) or that the base of the food web was more enriched in ^15^N following lower ice condition in the spring. Little evidence is suspected for baseline isotopic shifts in this system (Moody et al., 2012), but lack of representative samples of a secondary producer (e.g., *Calanus* spp.) or primary producer (e.g., phytoplankton) collected over multiple years during the ice melting and postmelting season in Lancaster Sound preclude us ignoring the hypothesis of possible changes in basal food web δ^15^N. For example, Søreide, Hop, Carroll, Falk‐Peterson, and Nøst Hegseth (2006) found lower δ^15^N in particulate organic matter (POM) from ice algae compared to pelagic water phytoplankton. Such differences suggest that depending on the phenology of ice breakup, and its associated ice algal bloom followed by a delayed pelagic primary productivity in the spring, secondary producers such as *C. glacialis* and *C. hyperboreus* might have access to different food sources of different quality (sympagic vs. pelagic) influencing their biomass development and growth (Leu, Søreide, Hessen, Falk‐Petersen, & Berge, 2011), and also their δ^15^N enrichment from a year to another.

Under heavy sea‐ice conditions, murres and kittiwakes had trophic positions similar to those of glaucous gulls. In that year, we also saw a shift toward higher δ^13^C values in eggs of murres, kittiwakes, and gulls. Ice algae are more enriched in ^13^C than particulate organic matter (POM) (Hobson, Ambrose, & Renaud, 1995; Hobson et al., 2002), and it is possible that this source of primary production was more important to food webs supporting seabirds in years of more extensive ice cover. This would also be consistent with higher δ^13^C values in eggs being the result of greater reliance on sympagic prey like Arctic cod by laying females (Budge et al., 2008; Hobson et al., 1995; Hobson & Welch, 1992a; Wang, Budge, Gradinger, Iken, & Wooller, 2014).  However, although sea ice is essential in structuring Arctic marine food webs, it can be restrictive through the barrier it creates with open water essential to foraging seabirds (Gaston, Gilchrist, & Hipfner, 2005; Gaston, Gilchrist, & Mallory, 2005). Increased isotopic overlap between murres and kittiwakes, due to the larger isotopic niche of the murres, could be reflecting the use of diverse prey types by the murres under the constraint of heavy sea ice.

When we considered a longer time series, there was no clear pattern between sea‐ice concentration and the isotopic niche of thick‐billed murre and fulmar, although isotopic niche areas of those species were variable among years. Even though sea‐ice concentrations did not relate to the species isotopic niche, both species occupied higher trophic positions with increased sea‐ice concentrations, which corroborated the results of Moody et al. (2012) for those species, but was opposite of what we observed for the gull and kittiwake in the four species model. Such influence of sea ice on trophic position likely indicates changes in the food web structure under various sea‐ice regimes (above; Norkko et al., 2007, Stabeno, Napp, Mordy, & Whitledge, 2010). Fulmars can travel long distances to forage (Mallory et al., 2008), such that they could exploit ocean and food conditions far from the colony. Although fulmars forage in Lancaster Sound, they have the ability to be going farther, and the consistency observed in fulmar niche area relative to sea‐ice concentration suggests they may be going where the influence of sea ice is limited (e.g., in Baffin Bay). Also, in contrast to murres, fulmars are surface feeders that generally depend on the food resources found in the top centimeters of the water column (but see Hobson & Welch, 1992b), which could restrict prey selection. Despite no distinct patterns in isotopic niche in relation to sea ice for the murre, its niche area was more variable, which clearly points to the different foraging behaviors adapted by murres and fulmars. Although unable to fly as far as the northern fulmar in a single foraging trip, being a surface diver, thick‐billed murres can exploit the water column vertically, accessing diverse prey type. Overall, the absence of relationship between the isotopic niche and sea ice for both murres and fulmars suggests a high degree of adaptation in foraging behavior that enables high Arctic seabirds to cope with unpredictable and likely restrictive conditions associated with variable sea‐ice cover.

We recognize that an isotopic niche is not necessarily an ecological niche and that the same isotopic values among individuals do not necessarily mean the same diet. To fully comprehend how sea‐ice dynamics influence the foraging ecology dynamics of top predators, investigating multiple aspects of community ecology is essential (e.g., prey samples, foraging behavior and bio‐logging, quantifying energy expenditure and metabolism). Stable isotopes are accessible tools to investigate trophic aspects within and sources of primary productivity to a community, and combined with other techniques, would enhance our overall comprehension of community dynamic under variable and changing environmental factors. Nonetheless, the isotopic space approach we have adopted provides a first look at potential variability in seabird foraging ecology related to sea ice in this system.

Collectively, we found that sea‐ice conditions affected the stable isotope values of prebreeding high Arctic seabirds and that this trophic response varied across species. The increased distance between the four species' isotopic niches suggests that reduction in sea‐ice cover might increase pressure on this Arctic seabird community, although this response could be due to effect size (only 3 years were used to assess the influence of sea‐ice conditions on the community isotopic niche). In that regard, the absence of a distinct relationship between isotopic niche and sea‐ice concentration when considering multiple years (murre and fulmar) stresses the relevance of investigating the community‐wide response to changes in the ocean and in sea‐ice conditions over time. Despite being subtle at the species level, we suggest that the effect of variable sea‐ice conditions might have larger implications when considering the trophic ecology of the community. Consequently, our study emphasizes the importance of considering a variety of organisms employing various foraging tactics (e.g., long‐distance forager, surface feeder, surface diver) to better use seabirds and generally marine top predators (Gulka, 2017), as indicators of ocean health and changes under the ecological pressure of a warming and more unpredictable climate.

## CONFLICT OF INTEREST

None declared.

## AUTHOR CONTRIBUTIONS

IP and MLM conceptualized the study, interpreted the results, and drafted the manuscript. IP analyzed the data. BB and KAH contributed with data and to laboratory analyses, revised, and improved the manuscript. All authors approved the final version of this manuscript.

## Data Availability

Isotopic data are available on Dryad https://doi.org/10.5061/dryad.46td5cq. Sea ice data: Sea ice concentrations from Nimbus‐7 SMMR and DMSP SSM/I passive microwave data—National Snow and Ice Data Center, Boulder, Colorado, USA, http://dx.doi.org/10.5067/8GQ8LZQVL0VL
